# 2023 FDA TIDES (Peptides and Oligonucleotides) Harvest

**DOI:** 10.3390/ph17020243

**Published:** 2024-02-13

**Authors:** Danah Al Shaer, Othman Al Musaimi, Fernando Albericio, Beatriz G. de la Torre

**Affiliations:** 1 Department of Medicinal Chemistry, Evotec (UK) Ltd., Abingdon OX14 4R, UK; danah.shaer@gmail.com; 2 School of Pharmacy, Faculty of Medical Sciences, Newcastle upon Tyne NE1 7RU, UK; 3 Department of Chemical Engineering, Imperial College London, London SW7 2AZ, UK; 4 School of Chemistry and Physics, University of KwaZulu-Natal, Durban 4001, South Africa; albericio@ukzn.ac.za; 5 CIBER-BBN, Networking Centre on Bioengineering, Biomaterials and Nanomedicine, Department of Organic Chemistry, University of Barcelona, 08028 Barcelona, Spain; 6 KRISP, College of Health Sciences, University of KwaZulu-Natal, Durban 4001, South Africa

**Keywords:** FDA approvals, drugs, peptides, oligonucleotides, avacincaptad pegol, eplontersen, nedosiran, tofersen, flotufolastat F, motixafortide, rezafungin, trofinetide, zilucoplan

## Abstract

A total of nine TIDES (pepTIDES and oligonucleoTIDES) were approved by the FDA during 2023. The four approved oligonucleotides are indicated for various types of disorders, including amyotrophic lateral sclerosis, geographic atrophy, primary hyperoxaluria type 1, and polyneuropathy of hereditary transthyretin-mediated amyloidosis. All oligonucleotides show chemically modified structures to enhance their stability and therapeutic effectiveness as antisense or aptamer oligomers. Some of them demonstrate various types of conjugation to driving ligands. The approved peptides comprise various structures, including linear, cyclic, and lipopeptides, and have diverse applications. Interestingly, the FDA has granted its first orphan drug designation for a peptide-based drug as a highly selective chemokine antagonist. Furthermore, Rett syndrome has found its first-ever core symptoms treatment, which is also peptide-based. Here, we analyze the TIDES approved in 2023 on the basis of their chemical structure, medical target, mode of action, administration route, and common adverse effects.

## 1. Introduction

**Figure 1:**
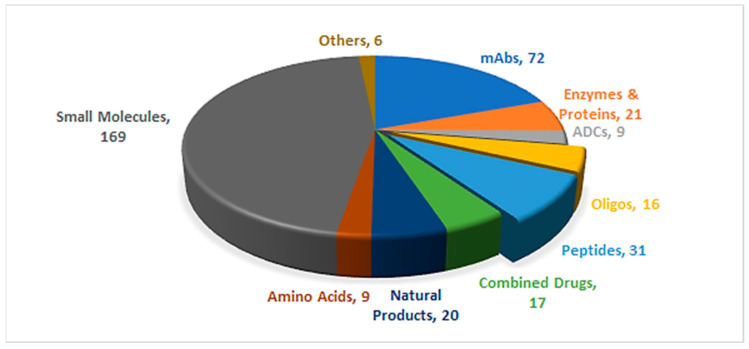
A total of 370 new drugs were approved by the Food and Drug Administration (FDA) between 2016 and 2023 [3]. ADCs, antibody–drug conjugates; mAbs, monoclonal antibodies; Oligos, oligonucleotides.

A total of 55 new chemical entities (NCEs) were approved by the Food and Drug Administration (FDA) during 2023 [1]. The approved number of drugs clearly demonstrates the recovery of the pharmaceutical industry sector after the disruption due to COVID-19 pandemic, where the agency returned to its approval pace (Figure 1) [2].

TIDES (peptides and oligonucleotides) made up 16% of the approved drugs in 2023, surpassing the approvals in 2022 (14%) [2]. The four oligonucleotides that received approval in 2023 had specific targets within various organs and body tissues, including the central nervous system, the eye, and the liver. Among these, two oligonucleotides were designed to target the liver, utilizing either the GalXC™ [4] or ESC-NGalNAc [5] platforms for delivery. These platforms employ GalNAc as a key ligand, facilitating interaction with the asialoglycoprotein receptor (ASGPR) in hepatocytes. The ligands are attached to the oligomer either as terminal dendrimers or as pendants on the monomers. Various structural peptides were approved in 2023, including linear, cyclic, and lipopeptides. Peptides consolidated their presence in the pharmaceutical arena, in which a peptide-based drug is considered the first-ever treatment for Rett syndrome. Doubtlessly, chemical modifications helped in boosting the pharmacokinetics and pharmacodynamics of peptides and bringing them to the market as effective therapies, rather than only focusing on endogenous human analogs [6]. Interestingly, and given the scarcity of the orally available peptides [2,7], an ultrashort tripeptide, trofinetide (Daybue^TM^), gained FDA approval to be administered orally. Noticeably, peptides are capable of selectively binding to a plethora of receptors either to activate or inhibit their functionality to achieve a desired therapeutic output [2]. Table 1 summarizes the 2023 FDA approvals of peptides and oligonucleotides.

## 2. Oligonucleotides

### 2.1. Tofersen (Qalsody^TM^)

**Figure 2:**
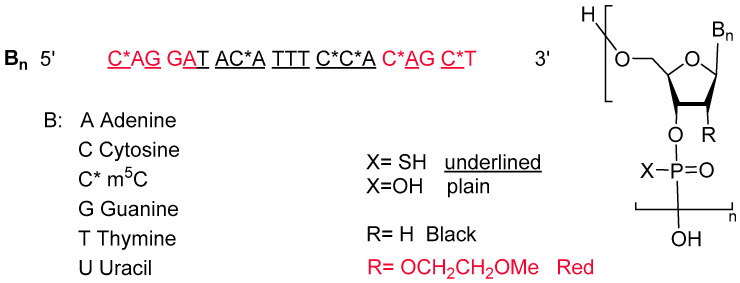
Chemical structure of Qalsody^TM.^

Tofersen is an antisense oligonucleotide comprising a chemically modified single-stranded RNA. It consists of 20 nucleosides with the majority of linkages (15 out of 19) being phosphorothioate bonds, where sulfur replaces the non-bridging oxygen of the phosphate group, enhancing the molecule’s stability. The 2′ position of the ribose unit of the last five monomers from each end is substituted with a methoxyethoxy group, while the ten residues in between have deoxygenated 2′ positions on the ribose unit. Additionally, all the Cytosine nitrogenous bases within the sequence are methylated at position 5 (m^5^C, Figure 2) [8,9].

Qalsody is prescribed for the management of amyotrophic lateral sclerosis (ALS) in adults with a mutation in the superoxide dismutase 1 (SOD1) gene [8]. ALS is an uncommon terminal neurodegenerative disorder marked by the progressive decline of motor neurons in the brain and spinal cord that control voluntary muscle movements. Although the exact cause of ALS is not well-defined, genetic and environmental factors are believed to play a role in the neurodegenerative process. SOD1 is a protein localized to the mitochondria and is crucial in protecting cells from damage caused by free radicals. Mutations in the SOD1 gene, among other mutations, contribute to the formation of misfolded proteins that accumulate in the neuron’s cytoplasm, ultimately leading to its death [9].

Tofersen binds to and silences the SOD1 mRNA, triggering its degradation, and subsequently reduces the synthesis of the SOD1 protein. This process slows down the progression of the disease [9].

It is administered intrathecally. The predominant adverse reactions, observed in more than 10% of patients receiving Qalsody and surpassing the placebo, encompass pain, fatigue, arthralgia, increased cerebrospinal-fluid white blood cell count, and myalgia.

Qalsody is produced by Biogen MA Inc and is licensed from Ionis Pharma [9]. It received FDA approval on 25 April 2023 [9]. This indication was granted accelerated approval, supported by the observed reduction in plasma neurofilament light chain levels in patients treated with Qalsody [8].

### 2.2. Avacincaptad Pegol (Izervay^TM^)

**Figure 3:**
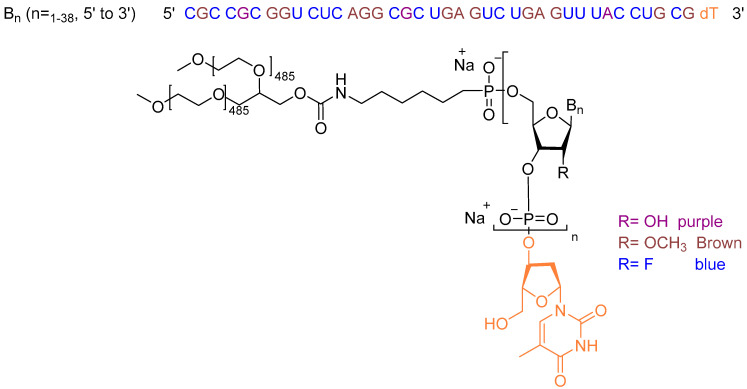
Chemical structure of Izervay^TM.^

Avacincaptad pegol is a sodium salt of a pegylated 39mer RNA aptamer ending with a deoxythymidine residue at the 3′ terminus. The thymidine residue is linked to the adjacent nucleoside through a 3′ to 3′ phosphodiester bond. The rest of the monomers throughout the chain are linked to each other via normal 5′ to 3′ phosphodiester linkages. Only three monomers have a hydroxyl group in position 2′ of the ribose unit, while the rest of the nucleosides undergo either methylation or fluoro-substitution, with the terminal thymidine being deoxygenated. The 5′ terminus of the oligonucleotide is attached to a double-branched polyethylene glycol chain through a carbamate group and a short alkylene spacer (Figure 3) [10].

Avacincaptad pegol is used for the treatment of geographic atrophy (GA) secondary to age-related macular degeneration (AMD) [10,11]. Geographic atrophy is the late stage of AMD, a condition which impairs the vision of patients due to the irreversible waste of cells in the macula in the eye. Degeneration of macular photoreceptors is thought to be linked to polymorphism of the gene coding of complement proteins. The complement cascade produces the membrane attack complex (C5b-9), which is responsible for cell lysis and death. Avacincaptad pegol interferes with the process by inhibiting the C5 complement protein from splitting into C5a and C5b fragments, two key components in the cascade [12], thus inhibiting the formation of the membrane attack complex and the subsequent cell lysis.

It is administered by intravitreal injections per eye per month for up to 12 months [11,12]. Avacincaptad pegol is well tolerated and some adverse effects observed were conjunctival hemorrhage, increased intraocular pressure (IOP), blurred vision, and neovascular age-related macular degeneration [10].

Avacincaptad pegol (Izervay™; formerly Zimura^®^) is developed by IVERIC Bio, an Astellas company [11], and was approved by the FDA on 4 August 2023 [11].

### 2.3. Nedosiran (Rivfloza^TM^)

**Figure 4:**
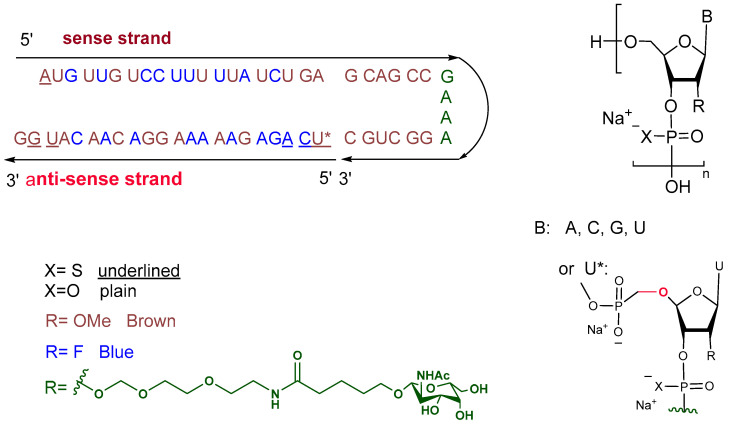
Chemical structure of Rivfloza^TM.^

Nedosiran is a small interference RNA (siRNA) that comprises the sodium salt of two chemically modified oligonucleotide chains. The sense strand, also known as the passenger, is a 36-mer and is longer than the antisense strand, which is a 22-mer and is referred to as the guide. Among the backbone linkages, the sense strand has 1 phosphorothioate out of 35 linkages and the antisense has 5 out of 21. All monomers of both strands are modified in position 2′ of the ribose unit. In addition to the most common OMe/F substitution in that position, four monomers of the sense strand contain N-acetyl-D-galactosamine substituents. These four monomers do not contribute to pairing and form a hair-pin-like secondary structure. On the other hand, the initial nucleoside in the antisense strand features a Uracil base and a modified ribose unit 5′ (Figure 4), as well as the 2′ position (methylated) [13]. Nedosiran uses the GalXC™ platform, in which the GalNAc targeting the asialoglycoprotein receptor (ASGPR) in hepatocytes is conjugated to the RNA chain [14]. This technology, owned by Dicerna Pharmaceuticals [4], resembles the ESC-NGalNAc platform for liver targeting drugs [5].

Nedosiran is indicated to lower urinary oxalate levels in children 9 years of age and older and adults with primary hyperoxaluria type 1 (PH1) [13].

Primary hyperoxaluria type 1 (PH1) is due to a shortage in the hepatic peroxisomal enzyme alanine-glyoxylate aminotransferase (AGT). AGT plays a crucial role in converting glyoxylate to glycine. In the absence of AGT activity, glyoxylate is transformed into oxalate by the lactate dehydrogenase enzyme (LDH; encoded by the LDHA gene in the liver), giving rise to insoluble calcium oxalate crystals that accumulate in organs, notably the kidneys, causing renal diseases [15]. Nedosiran binds to the RNA-induced silencing complex (RISC) and degrades the *LDHA* mRNA, lowering the production and precipitation of oxalate [14].

Nedosiran is the second siRNA to be approved for the treatment of PH1 following lumasiran, developed by Alnylam [16]. Both drugs reduce the production of oxalate but with different targets [14]. Lumasiran utilizes the ESC-NGalNAc platform developed by Alnylam [5].

Rivfloza is administered subcutaneously (SC) once per month, and its most common side effects are injection site reactions [13,14]. Rivfloza is developed by Dicerna Pharmaceuticals, a Novo Nordisk company, and was approved by the FDA on 29 September 2023 [14].

### 2.4. Eplontersen (Wainua^TM^)

**Figure 5:**
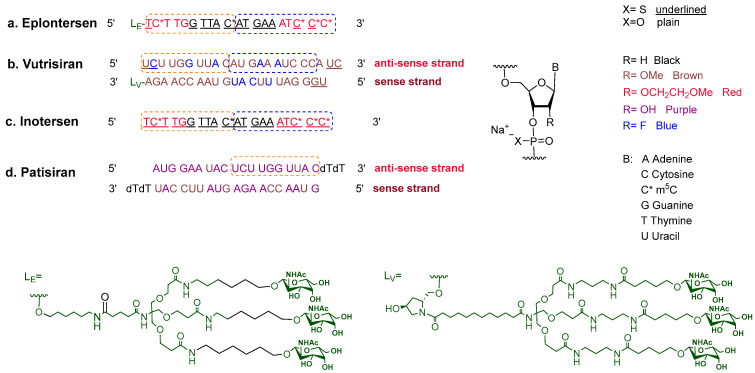
Chemical structures of (**a**) Wainua^TM^, (**b**) Amvuttra^TM^, (**c**)Tegsedi^TM^, and (**d**) Onpattro^TM.^

Eplontersen is a modified single-stranded antisense DNA chain consisting of 20-mer with 13 out of 19 of the phosphodiester linkages replaced with phosphorothioate bonds. All the Cytosine nitrogenous bases within the sequence are 5-methylated (m^5^C). The 2′-position of the ribose unit of the last five nucleosides on each side of the chain bears a methoxyethoxy substituent. A ligand (L_E_) bearing the N-acetylgalactosamine (N-AcGal) dendrimer is conjugated to the 5′ end of the chain (Figure 5a). The ligand L_E_ drives the oligonucleotide to the hepatocytes’ target [17]. Eplontersen is the fifth drug to employ Enhanced Stabilization Chemistry (ESC)-NGalNAc delivery technology to boost its PD/PK profiles. The technology was first introduced by Alnylam for liver targeting drugs in 2019 [5].

Eplontersen is indicated for the treatment of the polyneuropathy of hereditary transthyretin-mediated amyloidosis in adults (haTTR-PN) [18].

Transthyretin (TTR) is a tetramer protein that is produced in the liver and contributes to transferring thyroid hormone and retinol (vitamin A) in the body. Some mutations in the TTR gene, most commonly Val30Met, which replaces the Val residue in position 30 with a Met, destabilize the tetramer, boosting its dissociation and causing subsequent misfolding and aggregate accumulation in different tissues in the body and causing damage to the organs [19,20]. Eplontersen silences the TTR mRNA and suppresses the translation and misfolded protein, thus reducing the amyloidosis. Eplontersen is the fourth oligonucleotide drug for the treatment of haTTR after patisiran, inotersen [21], and vutrisiran [2]. Eplontersen shares a very similar sequence to inotersen and vutrisiran, and all three share a 10-mer segment (in the orange dashed box in Figure 5) with patisiran. It is worth noting that the only difference in the structure between eplontersen and inotersen is the presence of the liver targeting ligand conjugated to the former.

Previous treatments for haTTR were based on either liver transplant, which is hampered by the rare availability of a matching donor [22], or the use of tafadimis, a drug that binds to TTR and stabilizes the tetramer, slowing its dissociation [23]. The gene silencing technology showed better results in lowering the formation of amyloids over tafamidis treatment. However, low levels of vitamin A can be caused by the former and therefore supplements are recommended [22].

Wainua is administered SC once per month, and it is the first self-administered haTTR-PN drug via an auto-injector [18]. The most common side effects are vomiting and vitamin A depletion [18]. Wainua was developed under the AstraZeneca–Ionis Pharmaceuticals agreement, and was approved by the FDA on 21 December 2023 [18].

## 3. Peptides

### 3.1. Flotufolastat F 18 (Posluma^TM^)

**Figure 6:**
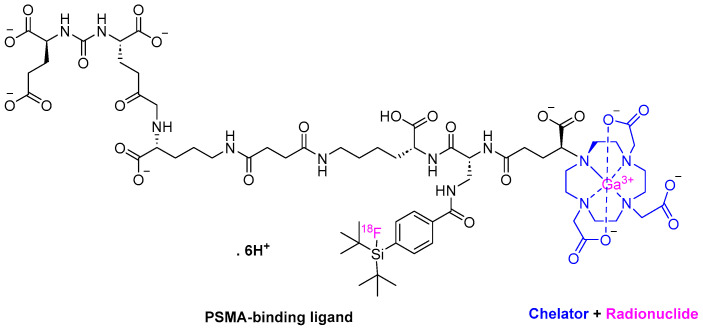
Chemical structure of Posluma^TM^.

Flotufolastat F 18 is a radioactive diagnostic agent that is composed of a prostate-specific membrane antigen (PSMA)-binding ligand. It is a DOTAGA complex with nonradioactive Ga^3+^ and a radioactive ^18^F covalently bound to silicon [24,25] (Figure 6). Posluma is used along with the positron emission tomography (PET) of PSMA-positive lesions in men with prostate cancer [25].

Flotufolastat F 18 binds to PSMA overexpressed by prostate cancer cells, and the complex is then internalized; ^18^F emits β+, which can be detected using PET [24,25]. ^18^F has shown several advantages over ^68^Ga, including better PET images ascribed to lower positron energy and a higher positron yield, in addition to a higher diagnostic accuracy as a result of its higher decay half-life (109.8 min) [26,27] versus that (68.6 min) for ^68^Ga [28].

Posluma is the forth drug of the same class to be approved for the same indication after Pluvicto [2], Pylarify [7], and ^68^Ga gozetotide [29], which were approved in 2022, 2021, and 2020, respectively. Interestingly, it is the first diagnostic agent to be approved with proprietary radiohybrid (rh) technology which offers two binding sites for radionuclides [30]. The technology was initially developed at the Technical University of Munich and then was out-licensed to Scintomics GmbH in 2017 [24]. Posluma showed high detection efficiency, even in patients with low PSMA levels [31,32], and its performance was comparable to the ^68^Ga-PSMA-11 (^68^Ga gozetotide) agent [31,32], which was approved in 2020 [29], as well as other PSMA ligands [33].

It is administered intravenously (IV) and has shown the following adverse effects, i.e., diarrhea, blood pressure increase, and injection site pain [25]. It was developed by Blue Earth Diagnostic, a subsidiary of Bracco Imaging, and received FDA approval on 25 May 2023 [24].

### 3.2. Motixafortide (Aphexda^TM^)

**Figure 7:**
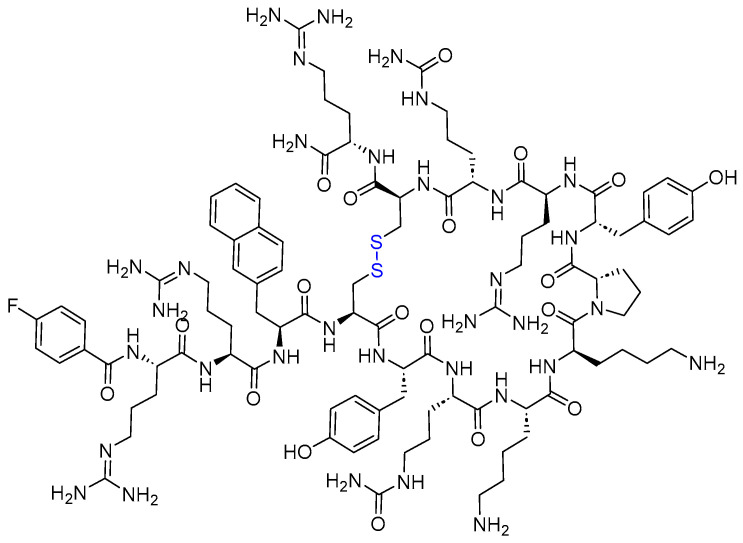
Chemical structure of Aphexda^TM^. Blue: disulfide bridge.

Motixafortide is a 14-mer cyclic peptide amide that acts as a selective chemokine receptor 4 (CXCR4) inhibitor. It comprises a disulfide bridge between Cys 4 and Cys 13 (blue) (Figure 7) [34]. Motixafortide is used with filgrastim (G-CSF) to mobilize hematopoietic stem cells (HSCs) in patients with multiple myeloma [35]. However, the FDA previously approved two small molecules (maraviroc and plerixafor) and one monoclonal antibody (mogamulizumab); motixafortide is the first peptide-based drug to be approved as a chemokine antagonist [36] and was granted an orphan drug designation [34]. It’s noteworthy that a cyclic peptide, balixafortide, functions similarly to motixafortide, but it fell short of meeting the co-primary endpoint related to the objective response rate (ORR) [36].

Motixafortide is a C-X-C Motif CXCR4 long acting inhibitor [37], which blocks the binding of its cognate ligand, stromal-derived factor-1α (SDF-1α)/C-X-C motif chemokine ligand 12 (CXCL12) [35].

Clinical studies are ongoing for the mobilization of CD34+ HSCs for gene therapy in patients with sickle cell disease [34,36]. In phase I, motixafortide was able to rapidly mobilize CD34+ cells and immune cells in healthy volunteers [37,38,39]. Motixafortide also showed promising data in pancreatic, breast, and lung cancers [40].

Motixafortide is administered intramuscularly (IM) and has the following adverse effects, i.e., injection site reactions, injection site pain, injection site erythema, injection site pruritus, pruritus, flushing, and back pain [35]. In fact, those adverse effects are considered mild to moderate. For example, transient injection site and systemic reactions were mitigated by methylprednisolone, paracetamol, and promethazine pretreatment [38]. Biokine Therapeutics acquired motixafortide from Kyoto University, and then the former entered into an exclusive worldwide license agreement with BioLineRx for its development and commercialization. It was approved by the FDA (in combination with filgrastim) on 08 September 2023 [34].

### 3.3. Rezafungin (Rezzayo^TM^)

**Figure 8:**
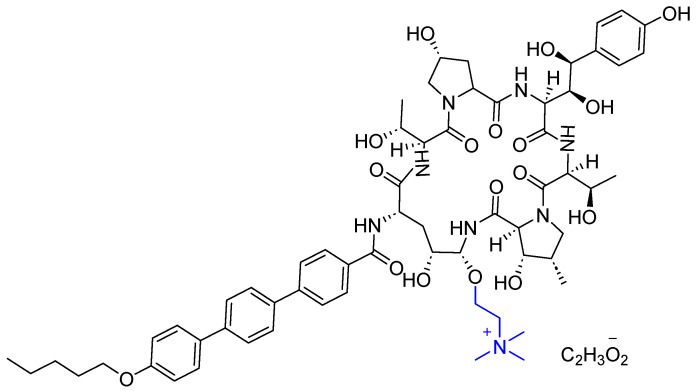
Chemical structure of Rezzayo^TM^. Difference from anidulafungin is shown in blue.

Rezafungin is a semisynthetic echinocandin antifungal lipopeptide used to treat patients 18 years of age or older who have limited or no alternative options for the treatment of candidemia and invasive candidiasis [41]. The FDA has approved three types of this class and they all share the same mechanisms of action, namely micafungin [42], anidulafungin [43], and caspofungin [44,45]. Rezafungin’s structure differs from that of anidulafungin by replacing the OH in anidulafungin with (trimethylammonio)ethoxy moiety (Blue) (Figure 8).

Rezafungin inhibits the 1,3-β-D-glucan synthase enzyme complex present in fungal cells, and hence inhibits the formation of 1,3-β-D-glucan which is an essential component of the fungal cell wall of many fungi, including *Candida species* (spp.) [45]. Rezafungin provides better tissue penetration, pharmacokinetics/pharmacodynamics, as well as a more tolerable safety profile than other echinocandin analogs [46]. The three phases of clinical studies were completed and proved that rezafungin is noninferior to caspofungin [47,48]. Day-30 mortality was the same (19%) between groups receiving either rezafungin or caspofungin, while mycological eradication occurring by day 5 was 73% in the rezafungin group and 65% in the caspofungin group, and the overall safety profile was the same across groups [49].

Currently, rezafungin is under phase 1 clinical trial to test its pharmacokinetics, safety, and tolerability in pediatric patients from birth to <18 years of age who are receiving concomitant systemic antifungal treatment as standard of care [41].

It is administered IV and showed several adverse effects, including hypokalemia, pyrexia, diarrhea, anemia, vomiting, nausea, hypomagnesemia, abdominal pain, constipation, and hypophosphatemia [45]. It was discovered by Seachaid, developed by Cidara Therapeutics Inc, licensed by Melinta Therapeutics LLC to commercialize it, and approved by the FDA on 22 March 2023 [41].

### 3.4. Trofinetide (Daybue^TM^)

**Figure 9:**
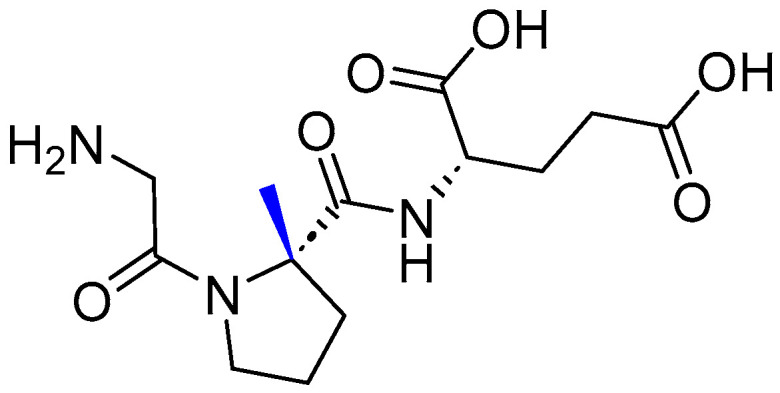
Chemical structure of Daybue^TM^. Difference from Glypromate is shown in blue.

Trofinetide is an α-methylated proline moiety analog to Gly–Pro–Glu, the *N*-terminal cleavage tripeptide product of the human insulin-like growth factor 1 (IGF-1) protein (Glypromate) (Figure 9) [50,51]. The α-methylation plays a central role in boosting the half-life of trofinetide as well as its bioavailability in comparison to its parent analog [51].

It is the first-ever treatment to treat Rett syndrome, which is a rare, genetic neurodevelopmental disorder [50]. Rett syndrome was initially described by Andreas Rett in 1966 [52] and is characterized by a loss of verbal communication with limited nonverbal skills, loss of fine and gross motor function (including purposeful hand use), behavioral issues, seizures, hand stereotypies, and gastrointestinal problems [53,54,55].

Trofinetide inhibits the production of inflammatory cytokines, inhibits the overactivation of microglia and astrocytes, and increases the amount of available IGF-1 that can bind to IGF-1 receptors [51]. After demonstrating the clinical benefit of trofinetide in a phase 2 study, phase 3 confirmed that trofinetide provides benefit in treating the core symptoms of Rett syndrome [50].

It is administered orally and has shown diarrhea and vomiting as common side effects [56]. It was discovered by Neuren, developed by Acadia Pharmaceuticals, and received FDA approval on 10 March 2023.

### 3.5. Zilucoplan (Zilbrysq^TM^)

**Figure 10:**
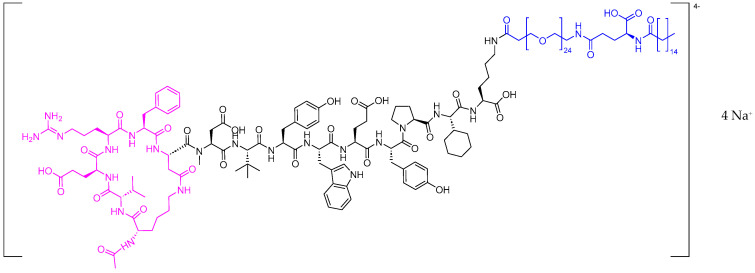
Chemical structure of Zilbrysq^TM^. Pink: lactam 1–6 cycle. Blue: N^ε^palmitoyl-γ-L-glutamyl.

Zilucoplan is a complement inhibitor cyclic peptide, lactam (1–6) (Pink), with a N^ε^palmitoyl-γ-L-glutamyl moiety pended from its *C*-terminal Lys (Blue) (Figure 10). It is used to treat generalized myasthenia gravis (gMG) in adult patients who are anti-acetylcholine receptor (AChR) antibody-positive [57]. gMG is a chronic, fluctuating, antibody-mediated autoimmune disorder directed against the post-synaptic neuromuscular junctions of skeletal muscles, resulting in a wide spectrum of manifestations ranging from mild to potentially fatal [58,59], with a localized or general weakness being the predominant symptom [59].

Zilucoplan specifically binds to complement protein C5, inhibits its cleavage to C5a (a potent anaphylatoxin) and C5b by C5 convertases, and prevents the formation of the cytolytic membrane attack terminal complement complex (MAC) or C5b9 [57,60]. Hence, zilucoplan acts within a dual mechanism to inhibit the formation of MAC, inhibiting the cleavage of C5, interfering with the formation of C5b6, and inhibiting red blood cell (RBC) hemolysis induced by plasmin-mediated non-canonical C5 activation [61,62]. Clinical studies demonstrated the efficacy of zilucoplan to inhibit the activation of C5, including wild-type and clinical R885 variants that do not respond to eculizumab treatment [60]. A phase 3 clinical study demonstrated the efficacy and safety of zilucoplan, in which patients who received zilucoplan showed a high reduction in myasthenia gravis activities of daily living (MG-ADL), while one patent died from each group (placebo and drug groups), which was not related to zilucoplan [63].

It is administered SC and has shown the following side effects, i.e., injection site reactions, upper respiratory tract infection, and diarrhea [57]. It was discovered by RA Pharmaceuticals, developed by UCB Pharma, and received FDA approval on 17 October 2023.

## 4. Conclusions

The successful story of therapeutic TIDES is progressing at a steady pace, with a total of 47 new TIDES drug entities approved in the period 2016–2023. The harmonization among various disciplines has indeed succeeded in tackling persistent obstacles in the way of drugs entering the market. The collaboration between academia and industry is fruitful in developing more robust drugs. For instance, the development journey of two of the approved peptides was initiated by academia, namely Aphexda^TM^ and Posluma^TM^, by Kyoto University and TU Munich, respectively. We are witnessing a real revolution in the various ways that TIDES molecules can be delivered with the desired purity, yield, activity, and at a large scale, too.

Oligonucleotides are gaining special attention from research and development companies, as they (the oligonucleotides) have revolutionized the pharmaceutical industry due to their potential to manage and treat rare genetic-related disorders. This is in addition to their capacity to perform chemical modifications without compromising their ability to bind to the complementary target, which confers large stability with good effectiveness.

Remarkably, a first-ever treatment for Rett syndrome was assigned for a peptide-based drug. In addition, peptides have joined small molecules and monoclonal antibodies to perform challenging tasks (mobilizing hematopoietic stem) with superior efficacy than the former and with a lower cost than the latter. Furthermore, unlike small molecules, peptides can act in a dual mechanism to exert their intended therapeutic action, ascribed to their large binding footprint, or interface with the therapeutic target. Balixafortide is another 15-mer cyclic peptide that showed promising data [64] and is currently being investigated for the same purpose by a phase 3 trial (NCT03786094) [36]. The aforementioned facts shed light on the ability of peptides to tackle persistent challenges and unmet medical needs.

As we pointed out in our previous reviews, we are actually witnessing increasing approvals of TIDES-based drugs with significant efficacies. Doubtlessly, these milestones are underpinned by the continuous efforts invested in developing their synthetic methodologies as well as the advanced understanding of the biological targets that aided the design process.

## Figures and Tables

**Table 1:** Summary of FDA-approved TIDES in 2023.

#	Active Ingredient (Trade Name)	Indication	Therapeutic Target	Administration Route
Oligonucleotides				
**1**	Tofersen (Qalsody^TM^)	To treat amyotrophic lateral sclerosis in adults who have an SOD1 gene mutation	Superoxide dismutase 1 (SOD1) mRNA	Intrathecally
**2**	Avacincaptad pegol (Izervay^TM^)	To treat geographic atrophy secondary to age-related macular degeneration	C5 complement protein	Intravitreally
**3**	Nedosiran (Rivfloza^TM^)	To lower urinary oxalate levels in patients 9 years and older with primary hyperoxaluria type 1 and relatively preserved kidney function	RNA-induced silencing complex (RISC)	Subcutaneously
**4**	Eplontersen (Wainua^TM^)	To treat polyneuropathy of hereditary transthyretin-mediated amyloidosis	Transthyretin (TTR) mRNA	Subcutaneously
Peptides				
**5**	Flotufolastat F-18 (Posluma^TM^)	To use with positron emission tomography (PET) imaging in certain patients with prostate cancer	Prostate-specific membrane antigen (PSMA)	Intravenously
**6**	Motixafortide (Aphexda^TM^)	To use with filgrastim (G-CSF) to mobilize hematopoietic stem cells to the peripheral blood for collection and subsequent autologous transplantation in patients with multiple myeloma	Chemokine receptor 4 (CXCR4)	Intramuscularly
**7**	Rezafungin (Rezzayo^TM^)	To treat candidemia and invasive candidiasis	1,3-β-D-glucan synthase enzyme	Intravenously
**8**	Trofinetide (Daybue^TM^)	To treat Rett syndrome	Inflammatory cytokines	Orally
**9**	Zilucoplan (Zilbrysq^TM^)	To treat generalized myasthenia gravis in adults who are anti-acetylcholine receptor (AChR) antibody-positive	C5 protein	Subcutaneously
